# Azithromycin effectiveness against intracellular infections of *Francisella*

**DOI:** 10.1186/1471-2180-10-123

**Published:** 2010-04-23

**Authors:** Saira Ahmad, Lyman Hunter, Aiping Qin, Barbara J Mann, Monique L van Hoek

**Affiliations:** 1 Department of Molecular and Microbiology, National Center for Biodefense and Infectious Diseases, George Mason University, Manassas, VA, 20120, USA; 2 Inova Fairfax Hospital, Falls Church, VA 22042, USA; 3 Departments of Medicine & Microbiology, University of Virginia Health Systems, Charlottesville, VA 22908, USA

## Abstract

**Background:**

Macrolide antibiotics are commonly administered for bacterial respiratory illnesses. Azithromycin (Az) is especially noted for extremely high intracellular concentrations achieved within macrophages which is far greater than the serum concentration. Clinical strains of Type B *Francisella *(*F.*) *tularensis *have been reported to be resistant to Az, however our laboratory *Francisella *strains were found to be sensitive. We hypothesized that different strains/species of *Francisella *(including Type A) may have different susceptibilities to Az, a widely used and well-tolerated antibiotic.

**Results:**

*In vitro *susceptibility testing of Az confirmed that *F. tularensis subsp. holarctica *Live Vaccine Strain (LVS) (Type B) was not sensitive while *F. philomiragia, F. novicida*, and Type A *F. tularensis *(NIH B38 and Schu S4 strain) were susceptible. In J774A.1 mouse macrophage cells infected with *F. philomiragia, F. novicida*, and *F. tularensis *LVS, 5 μg/ml Az applied extracellularly eliminated intracellular *Francisella *infections. A concentration of 25 μg/ml Az was required for *Francisella-*infected A549 human lung epithelial cells, suggesting that macrophages are more effective at concentrating Az than epithelial cells. Mutants of RND efflux components (*tolC *and *ftlC*) in *F. novicida *demonstrated less sensitivity to Az by MIC than the parental strain, but the *tolC *disc-inhibition assay demonstrated increased sensitivity, indicating a complex role for the outer-membrane transporter. Mutants of *acrA *and *acrB *mutants were less sensitive to Az than the parental strain, suggesting that AcrAB is not critical for the efflux of Az in *F. novicida*. In contrast, *F. tularensis *Schu S4 mutants Δ*acrB *and Δ*acrA *were more sensitive than the parental strain, indicating that the AcrAB may be important for Az efflux in *F. tularensis *Schu S4. *F. novicida *LPS O-antigen mutants (*wbtN, wbtE, wbtQ *and *wbtA*) were found to be less sensitive *in vitro *to Az compared to the wild-type. Az treatment prolonged the survival of *Galleria *(*G*.) *mellonella *infected with *Francisella*.

**Conclusion:**

These studies demonstrate that Type A *Francisella *strains, as well as *F. novicida *and *F. philomiragia*, are sensitive to Az *in vitro. Francisella *LPS and the RND efflux pump may play a role in Az sensitivity. Az also has antimicrobial activity against intracellular *Francisella*, suggesting that the intracellular concentration of Az is high enough to be effective against multiple strains/species of *Francisella*, especially in macrophages. Az treatment prolonged survival an *in vivo *model of *Francisella-*infection.

## Background

Bacteria in the *Francisella *genus are nonmotile, nonsporulating, gram-negative coccobacilli. *Francisella *causes a zoonotic disease; humans can become infected via a variety of mechanisms including inhalation of an extremely low infectious dose [1]. *F. tularensis *primarily targets macrophages where bacterial survival and replication occurs [1]. The genus *Francisella *is divided into two species: *tularensis *and *philomiragia*. *Francisella tularensis *has four subspecies: *F. tularensis *subspecies *tularensis *(formerly *F. tularensis*,) *F. tularensis *subspecies *holarctica *(which includes the live vaccine strain, LVS), *F. tularensis *subspecies *mediasiatica*, and *F. tularensis *subspecies *novicida *(*F. novicida*) [2]. Subspecies of *Francisella tularensis *are further separated into two types depending on their virulence. Type A strains include *Francisella tularensis *subspecies *tularensis *Schu S4 (*F. tularensis *Schu S4) and are more virulent [3], except for the ATCC type strain *F. tularensis *subsp. *tularensis *NIH B38 which is avirulent [4-6]. *Francisella *Type A strains are normally associated with ticks and rabbits and are restricted to North America. Type B strains (*Francisella tularensis *subspecies *holarctica *and *mediasiatica*) are less virulent and cause tularemia throughout Eurasia [3].

Standard recommended antibiotic treatment for tularemia includes oral tetracycline antibiotics (e.g. doxycycline) and fluoroquinolones (e.g. ciprofloxacin) which have adverse side-effects on pediatric and the elderly patients, and individuals with liver disease. Aminoglycosides such as streptomycin and gentamicin can be injected intravenously or intramuscularly [7], but are not commonly used. Macrolides are oral antibiotics commonly used to treat bacterial respiratory illnesses. Azithromycin (Az), a member of the azalide subclass of macrolides, binds to the 50 s subunit of gram-negative bacterial ribosomes, and inhibits translation of mRNA resulting in inhibition of bacterial growth or death [8]. It has been suggested that the two basic amine sites of Az interact with the negatively charged heptose-phosphate region of lipopolysaccharide (LPS) in order to enter gram-negative bacteria [9]. *F. novicida *transposon insertion mutants in the genes involved in lipopolysaccharide (LPS) production (*wbtN, wbtE, wbtQ *and *wbtA*) were tested to determine if there might be a role of LPS in Az binding and penetration. Mutations in genes responsible for the synthesis of the O-antigen in *F. novicida *have been previously shown to decrease virulence and resistance to serum killing while macrophage uptake and replication remained unaffected [10].

**Figure 1:**
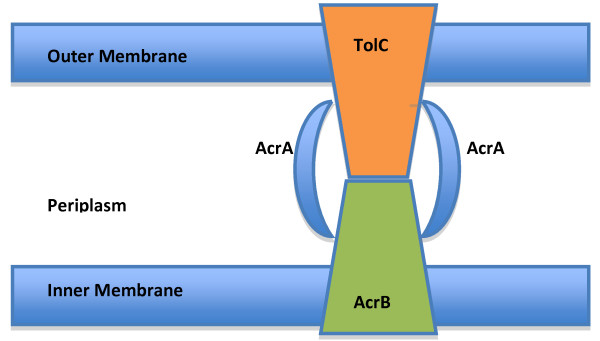
**RND efflux pump**. A schematic of the RND efflux pump, following [59], to illustrate the relationship between TolC, AcrA and AcrB.

A primary mode of bacterial resistance to antibacterial drugs is the expression of drug efflux pumps such as ATP-binding cassette (ABC), the Major Facilitator Superfamily (MFS) transporters, and Resistance-Nodulation-Division (RND) efflux system. These inner membrane transport systems are often coupled to the outer membrane TolC system [11]. *Francisella novicida *has two tolC-like proteins, *tolC *and the highly related *fltC *[12]. The ABC Superfamily is thought to be responsible for the export of many different antibiotics. For example, in *E. coli*, macrolides are thought to be transported by the ABC transporter MacAB [13]. Although a potential *macA *gene was identified in *F. novicida *(FTN_1692), no gene corresponding to *macB *could be identified in the *F. novicida *genome. The RND efflux system consists of a tripartite transporter with an RND pump protein located in the cytoplasmic membrane (AcrB) and a periplasmic membrane fusion protein (AcrA) coupled to the TolC protein in the outer membrane (Figure 1). The RND system can pump many compounds, including macrolides [14]. The AcrAB RND efflux pump was recently demonstrated to be required for *F. tularensis *LVS virulence in mice [15], but not in *F. tularensis *Schu S4 [16]. The function of the RND efflux system is the removal of harmful substances from inside the cytosol of the bacteria directly to the external medium bypassing the periplasm [15]. Thus we hypothesized that mutants in the RND efflux system would have altered sensitivity to Az. Transposon insertion mutants of components of the RND efflux system in *F. novicida*, including *tolC, fltC, acrA*, and *acrB*, were tested for their sensitivity to Az. The *dsbB *gene encodes the cytoplasmic membrane protein that is involved in disulfide bond formation in the periplasm. A *dsbB *mutant in *F. novicida *was tested because it is transcriptionally linked in an operon with *acrA *and *acrB *in *Francisella*. Mutants Δ*acrA *and Δ*acrB *were also tested in the fully virulent strain, *F. tularensis *Schu S4 [16].

Az, which is commonly prescribed to pediatric patients for treatment of common upper respiratory track and ear infections [17], has low toxicity and few side-effects [18]. When administered, the antibiotic becomes ion-trapped in the acidic lysosomes of white blood cells including macrophages resulting in a high intracellular concentration compared to the plasma during the dose period. Intracellular concentrations remain high after the dose period ends with a half-life of 68 hours [18].

Murine macrophages J774A.1 are a well-studied *in vitro *model system for tularemia [19,20] and were chosen as a model cell system to study *Francisella *infection and treatment by Az. The murine macrophage cell line J774A.1 supports the intracellular replication of *F. tularensis *LVS [19], *F. novicida *[21], and *F. tularensis *Schu S4 [16]. For a model of the human system, human lung epithelial cells A549 were chosen. *F. tularensis *LVS has been previously shown to infect and replicate within A549 cells [22-24]. We hypothesized that the ability of Az to concentrate at high levels within the macrophages may result in effectiveness against intracellular infections by *Francisella *species, even at extracellular Az levels lower than the MIC.

The larval stage of *Galleria *(*G.*)*mellonella*, wax moth caterpillar, has been used as a model to study infections caused by some bacteria including *F. tularensis *LVS [25]. The larvae do not have an adaptive immune system, but have resistance to microbial infections via cellular and humoral defenses [26]. The analysis of insect responses to pathogens can provide an accurate indication of the mammalian response to that pathogen. Physical effects such as color change can be observed when the bacteria replicates and increases in the larvae [25]. We used *G. mellonella *as an alternative to the mouse model of *Francisella *infection to test our hypothesis that Az treatment could prolong the survival of *Francisella *infected caterpillars.

## Results

### *Francisella's *sensitivity to Az

It has been reported that European clinical strains of Type B *F. tularensis *are resistant to Az [27]. However, we observed that commonly used laboratory strains of *Francisella *are sensitive to Az. *In vitro *susceptibility testing of Az confirmed that *F. tularensis *LVS strain was not highly sensitive *in vitro *to this antibiotic, confirming that the Type B strains are relatively resistant to this antibiotic. Our study demonstrated that *F. philomiragia, F. novicida *and Type A *F. tularensis tularensis*, including both *F. tularensis tularensis *NIH B38 and *F. tularensis *Schu S4 strains, were susceptible to this drug *in vitro *and *in vivo*.

*Francisella *strains were tested in a Kirby-Bauer disc inhibition assay for sensitivity to Az. *F. novicida, F. philomiragia*, and *F. tularensis tularensis *B38 were sensitive to 15 μg Az discs, whereas *F. tularensis *LVS was not sensitive to this concentration. *F. novicida *had a zone of inhibition of 28.7 ± 0.7 mm in diameter around the 6 mm Az disc, and *F. philomiragia*'s zone of inhibition was 21.7 ± 0.8 mm in diameter. *F. tularensis tularensis *NIH B38 had the largest zone of inhibition, 45.9 ± 6.2 mm in diameter around the Az disc (Table 1). These results were all significantly different than *F. tularensis *LVS (p-value < 0.001). Although *F. tularensis tularensis *NIH B38 is not virulent, this result suggested the potential sensitivity of the Type A strains to Az. In order to corroborate this with the fully virulent strain, *F. tularensis *Schu S4 was tested and determined to have a zone of inhibition of 25.5 ± 1.9 mm (p-value < 0.001 compared to *F. tularensis *LVS).

**Table 1:** Az Disk Inhibition Assay with *Francisella *strains.

Bacterial Strains	Antibiotic Zone of Inhibition (mm) (Disc is 6 mm)	p-value
*F. tularensis LVS*	6.0 ± 0	----

*F. novicida*	28.7 ± 0.7	<0.001

*F. philomiragia*	21.7 ± 0.8	<0.001

*F. tularensis NIH B38*	45.9 ± 6.2	<0.001

*F. tularensis *Schu S4	25.5 ± 1.9	<0.001

15 μg Az discs (Fluka) were placed on the agar and the zone of inhibition was measured. P-value was calculated compared to *F. tularensis *LVS.

**Figure 2:**
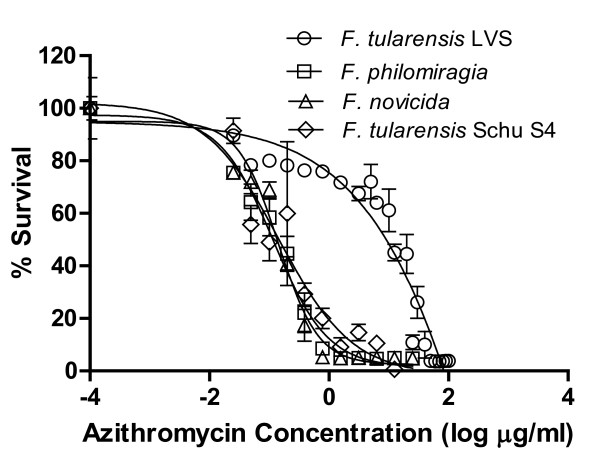
**MIC determination of Az for *F. tularensis *LVS, *F. philomiragia, F. novicida*, and *F.tularensis *Schu S4**. Az MIC for *F. tularensis *LVS (circles) is higher than *F. philomiragia *(squares), *F. novicida *(up triangle), and *F. tularensis *Schu S4 (down triangle). Az MICs for *F. novicida *and *F. tularensis *Schu S4 are 0.78 μg/ml with an EC_50 _of 0.16 μg/ml and 0.15 μg/ml respectively. *F. philomiragia*'s Az MIC is 1.56 μg/ml with an EC_50 _of 0.13 μg/ml, and *F. tularensis *LVS's Az MIC is 25 μg/ml with an EC_50 _of 17.34 μg/ml.

The Minimal Inhibitory Concentrations (MIC) for Az and gentamicin were measured in liquid broth assays to determine *Francisella *sensitivity to Az compared to control antibiotic gentamicin. *F. novicida *and *F. philomiragia *were more susceptible to Az than *F. tularensis *LVS, which was only susceptible to Az at higher concentrations. The MIC of Az for *F. novicida *is 0.78 μg/ml (EC_50 _of 0.16 μg/ml), and 1.56 μg/ml (EC_50 _of 0.13 μg/ml) for *F. philomiragia*. These results were all significantly different than *F. tularensis *LVS (MIC of 25.0 μg/ml; EC_50 _of 17.3 μg/ml; p-value ≤ 0.004) (Figure 2, Table 2). The MIC result for *F. tularensis *LVS explains why there was no inhibition of growth in the disc-diffusion assay, as there was only 15 μg of Az in the disc, which is below the MIC and the EC_50_. Our studies were performed with *Francisella *LVS strain NR-646 from BEI Resources, who state that it has been confirmed by PCR amplification of a sub-species specific sequence to be subsp. *holarctica *(Type B). Our results differ from those reported by Ikaheimo *et al. *for the Type B ATCC 29684, deposited in BEI as *Francisella *LVS NR-14, who reported a MIC for azithromycin of >256 mg/L [27]. Results for *F. tularensis *Schu S4 were similar to *F. novicida *with a MIC of 0.78 μg/ml, and EC_50 _of 0.15 μg/ml Az (Table 2). This is consistent with the disc inhibition assay results. These results are also similar to results with related macrolide antibiotic, erythromycin, which has a reported MIC of 0.5-4, and EC_50 _of 2 μg/ml against Type A and B *Francisella *strains, though not LVS (MIC > 256 μg/ml) [28]. As a control, we determined the MIC for the antibiotic gentamicin to which all strains of *Francisella *are susceptible [29]. The MIC of gentamicin for *F. novicida *was determined to be 0.2 μg/ml (EC_50 _of 0.12 μg/ml); for *F. philomiragia *the MIC was 0.39 μg/ml (EC_50 _of 0.22 μg/ml); and for *F. tularensis *LVS the MIC was 0.39 μg/ml (EC_50 _of 0.09 μg/ml) (Table 2). These values are consistent with published sensitivities of Type B strains (MIC of 0.03-0.5 μg/ml, EC_50 _of 0.12 μg/ml) [28]. Thus, the Type A *Francisella tularensis *SchuS4, *F. novicida *and *F. philomiragia *are all sensitive to Az *in vitro*. Type B *Francisella *LVS was also determined to be sensitive, but at a higher concentration of Az.

**Table 2:** MIC Assay of Az for *Francisella *strains.

Bacteria	Az MIC (μg/ml)	**Az EC**_**50**_**(μg/ml)**	p-value	Gent MIC (μg/ml)	**Gent EC**_**50**_**(μg/ml)**
*F. tularensis *LVS	25	17.34	----	0.39	0.09

*F. philomiragia*	1.56	0.13	<0.001	0.39	0.22

*F. novicida*	0.78	0.16	<0.001	0.20	0.12

*F. tularensis *Schu S4	0.78	0.1453	0.004	n/a	n/a

The p-value is for comparisons of the EC_50 _values.

**Figure 3:**
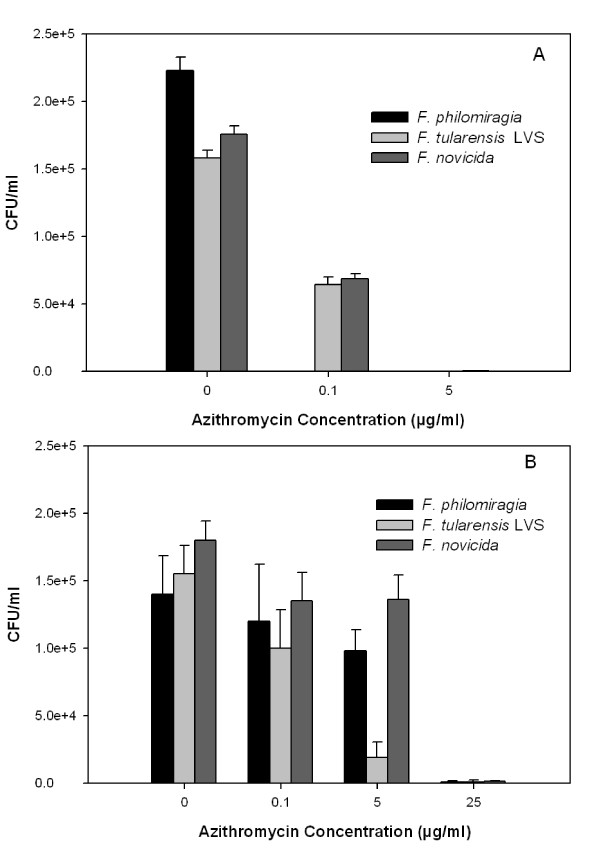
**Az inhibition of intracellular *Francisella *strains**. After 22 hours, recovered bacterial counts were measured for *F. philomiragia, F. novicida*, and *F. tularensis *LVS infected cells (MOI 500). A) J774A.1 cells infected with *F. philomiragia, F. novicida*, or *F. tularensis *LVS had more than 10^5 ^CFU/ml. Bacterial counts decreased for all strains as the Az concentrations increased and were near 0 CFU/ml at 5 μg/ml Az. B) A549 cells infected with *F. philomiragia*, *F. novicida*, or *F. tularensis *LVS had more than 10^5 ^CFU/ml at 0 μg/ml Az. Bacterial counts decreased at 0.1 and 5 μg/ml Az and were near 0 CFU/ml at 25 μg/ml Az. CFU counts from no Az treatment compared 0.1, 5, and 25 μg/ml Az treatment for all *Francisella *strains were significantly different (p-value < 0.005).

J774A.1 and A549 cells were infected with *Francisella *and treated with Az. The same multiplicity of infection (MOI = 500) was used, based on previous studies for *Francisella *infection [30]. Cells were lysed and bacteria were recovered and counted as colony forming units (CFU). *Francisella-*infected J774A.1 and A549 cells were found to have more than 10^5 ^CFU/ml of *Francisella *after 22 hours after infection. J774A.1 cells infected with *Francisella *and treated with Az had decreasing CFUs as the antibiotic concentration increased. In J774A.1 cells infected with *F. philomiragia*, no CFUs were recovered when treated with 0.1 μg/ml Az (less than the MIC). In J774A.1 cells infected with either *F. novicida *or *F. tularensis *LVS, bacterial concentrations decreased with the addition of Az. At 5 μg/ml Az, no CFUs were recovered (p-value < 0.005 compared to 0 μg/ml Az) (Figure 3A). In this case, the Az concentration was less than the MIC for *F. tularensis *LVS. *Francisella-*infected A549 cells required higher concentrations of Az than J774A.1 cells, suggesting that epithelial cells are not able to concentrate Az in the same manner as macrophages. As before, intracellular *F. novicida, F. philomiragia*, and *F. tularensis *LVS CFU counts decreased when A549 cells were treated with Az. Recovered intracellular CFU counts for *F. philomiragia *and *F. novicida *remained approximately equal when treated with 0.1 and 5 μg/ml Az (p-value > 0.05), but strongly decreased at 25 μg/ml Az (p-value < 0.005 compared to 0 μg/ml Az). For these two organisms, the required external antibiotic concentration was higher than the *in vitro *MIC. *F. tularensis *LVS infected A549 cells had a steady decline of intracellular CFU counts as the Az concentration increased and had essentially no colonies recovered at 25 μg/ml extracellular Az (p-value < 0.005 compared to 0 μg/ml Az), which is equivalent to the MIC for that strain (Figure 3B). The difference between the cell types may reflect the fact that J774A.1 cells are phagocytic macrophages, and the A549 cells are non-phagocytic epithelial cells.

To determine if *Francisella *bacteria counts were decreased due to Az concentrations or due to cell death, cellular lysis and apoptosis were measured by LDH released [19]. At 22 hours, cell cytotoxicity in non-infected A549 cells and A549 cells infected with *F. novicida, F. philomiragia*, and *F. tularensis *LVS remained below 20%. Non-infected A549 cells along with *F. philomiragia, F. novicida*, and *F. tularensis *LVS-infected cells had a slightly increased cytotoxicity as Az concentrations increased (Table 3). Cellular apoptosis remained low with all Az doses. These results suggest the decreased *Francisella *counts were due to Az treatment and not due to bacterial release during the experiment from apoptosis or cell lysis.

**Table 3:** A549 cell cytotoxicity.

Bacteria	0 μg/ml Az	0.1 μg/ml Az	1.0 μg/ml Az	2.5 μg/ml Az	5.0 μg/ml Az
A549 cells	0 ± 3.0	2.9 ± 2.8	8.0 ± 4.0	18.3 ± 5.2	19.7 ± 9.6

*F. novicida*	0 ± 2.3	4.1 ± 5.0	3.3 ± 6.3	9.6 ± 5.4	17.8 ± 13.2

*F. philomiragia*	0 ± 1.3	0 ± 2.5	7.1 ± 4.6	1.7 ± 3.2	8.5 ± 4.1

*F. tularensis *LVS	0 ± 3.7	2.12 ± 5.0	4.6 ± 5.9	8.4 ± 5.1	5.2 ± 5.6

Using a LDH release assay, the cell cytotoxicity as a result of antibiotic and/or *Francisella *infection was determined and is indicated as a percentage (%) of total LDH released.

### *Francisella *LPS mutants

**Figure 4:**
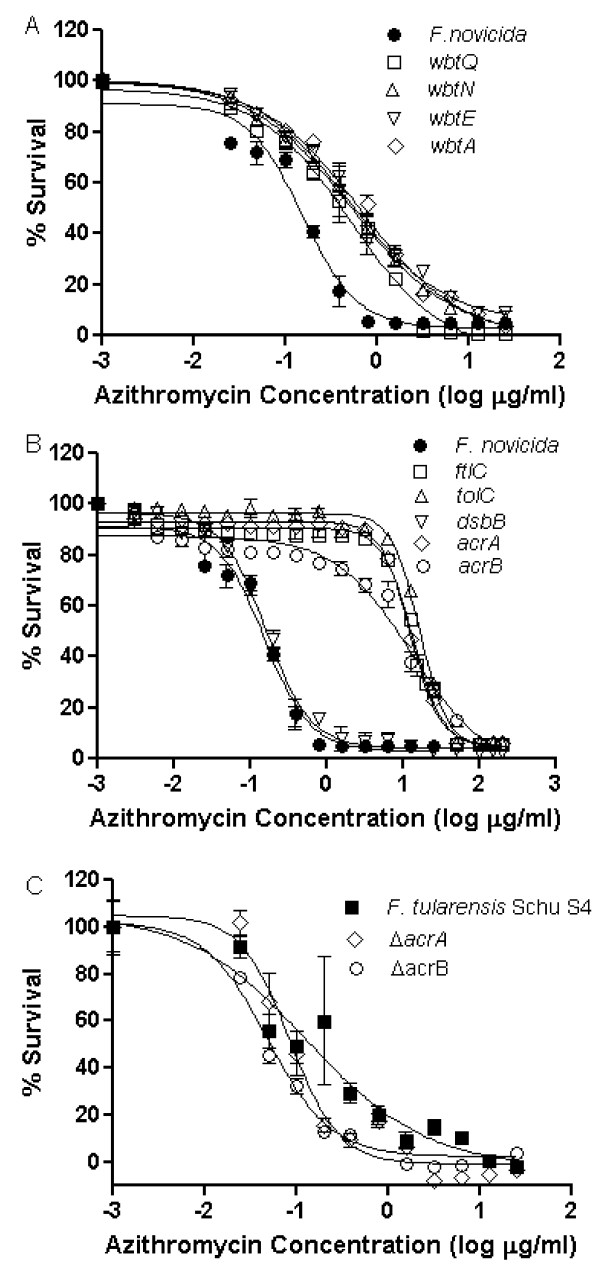
**MIC determination of Az for *F. novicida *transposon LPS and RND efflux mutants and *F. tularensis *Schu S4 RND efflux mutants**. A) The MIC of Az for LPS O-antigen *F. novicida *transposon mutants was generally higher than the wild-type (circle) MIC of 0.78 μg/ml. MICs for LPS O-antigen mutants were 12.5 μg/ml for *wbtA *(diamond), 25.0 μg/ml for *wbtE *(down triangle), 3.12 μg/ml for *wbtQ *(square), and 12.5 μg/ml for *wbtN *(triangle), with an EC_50 _for all LPS O-antigen mutants greater than 0.50 μg/ml (p-value < 0.005). B) MICs for *F. novicida *transposon-insertion RND efflux mutant varied: *dsbB *(down triangle) was closer to the wild-type (closed circle) at 1.56 μg/ml (p-value 0.400). *ftlC, tolC, acrA*, and *acrB *have greater MIC with 25 μg/ml for *ftlC *(square) and 50 μg/ml for *tolC *(up triangle), *acrA *(diamond), and *acrB *(open circle), with EC_50 _greater than 12 μg/ml (p-value < 0.005). C) The MICs of Az for *F. tularensis *Schu S4 (square) and deletion RND efflux mutants. *F. tularensis *Schu S4 (square) has an MIC of 0.78 μg/ml, Δ*acrB *(circle) of 1.56 μg/ml, and Δ*acrA *(diamond) of 3.13 μg/ml. *F. tularensis *Schu S4 and mutants all have EC_50 _less than 0.15 μg/ml (p-value < 0.1 for Δ*acrA *and Δ*acrB *compared to wild-type).

Due to the potential for interaction of Az with LPS [9], four *F. novicida *transposon LPS O-antigen mutants were tested for their Az susceptibility: O-antigen of LPS (*wbtA*) biosynthesis of GdNAcAN, an O-antigen unit (*wbtE*), glycosylatransferase that elongates to form GalNAcAN tri-saccharides (*wbtQ*), and aminotransferase (*wbtN*) [10]. *F. novicida *LPS O-antigen mutants including *wbtA, wbtE, wbtQ*, and *wbtN *were shown to be less susceptible to Az by decreased zones of inhibition in comparison to the wild-type (p-value < 0.001) (Table 4). The MICs for Az against the *F. novicida *LPS-related transposon mutants *wbtA, wbtE, wbtQ*, and *wbtN *(MIC's > 3.0 μg/ml Az, EC_50 _> 0.50 μg/ml Az) were greater than the wild-type MIC (0.78 μg/ml) (p-value < 0.005) (Figure 4A, Table 5), suggesting increased resistance to Az. These data are consistent with the disc inhibition studies, suggesting that *Francisella *LPS plays some role in the sensitivity of the strains for Az.

**Table 4:** Az Disk Inhibition Assay with *Francisella *transposon mutants of LPS production genes.

	Antibiotic No Growth Zone (mm)	
***F. novicida***	**Avg**	**P-value**

wild-type	28.7 ± 0.7	-------

*wbtA*	20.8 ± 0.5	<0.001

*wbtN*	23.3 ± 0	<0.001

*wbtE*	23.0 ± 0.9	<0.001

*wbtQ*	20.1 ± 1.3	<0.001

15 ug Az discs from Fluka were placed on an agar plate spread with the indicated strain. The zone of inhibition was measured in mm.

**Table 5:** MIC Assay of Az for *F. novicida *transposon mutants.

Bacteria	AZ MIC (μg/ml)	**AZ EC**_**50**_**(μg/ml)**	p-value
***F. novicida***	0.78	0.16	------

*wbtQ*	3.12	0.52	0.005

*wbtN*	12.5	0.54	<0.002

*wbtE*	25	0.50	<0.001

*wbtA*	12.5	0.67	0.007

*dsbB*	1.56	0.16	0.401

*ftlC*	25	13.47	<0.002

*tolC*	50	16.44	<0.001

*acrA*	50	12.39	<0.001

*acrB*	50	13.23	0.001

***F. tularensis *Schu S4**	0.78	0.1453	-------

Δ*acrA*	3.13	0.0852	0.087

Δ*acrB*	1.56	0.0493	0.031

MIC and EC_50 _were calculated as described. p-values compare the EC_50 _of mutants to wild-type *F. novicida *and *F. tularensis *Schu S4.

### *Francisella *RND mutants

Five *F. novicida *transposon insertion mutants in the multidrug efflux protein genes (*acrA *and *acrB*), the transcriptionally linked protein gene (*dsbB*), as well as the related outer membrane channel genes (*tolC *and *ftlC*) were tested to determine if Az susceptibility increases or decreases [12]. Results for the RND efflux mutants varied among the different subspecies (Table 6). In the disc inhibition assay, the *tolC *mutant was slightly more sensitive to Az compared to the wild-type *F. novicida *(p-value = 0.007), while *ftlC, acrA*, and *acrB *were less susceptible to Az compared to the wild-type (p-value < 0.01) (Table 6). The MICs for *ftlC*, *tolC*, *acrA*, and *acrB *(MIC = 25 μg/ml Az) were greater than the wild-type (MIC of 0.78 μg/ml Az) and had a higher EC_50 _(EC_50 _> 12 μg/ml Az) compared to the wild-type of 0.16 μg/ml Az (p-value < 0.002), indicating decreased sensitivity to the antibiotic. These results are consistent between the MIC and disc inhibition assay for *acrA, acrB*, and *ftlC *(Figure 4B, Table 5). The *tolC *sensitivity to Az results in the solid agar and liquid broth assay were inconsistent. The disc-inhibition assay suggests increased sensitivity, while the MIC assay demonstrated increased resistance. We are currently investigating the basis of this difference.

**Table 6:** Az Disk Inhibition Assay with *Francisella *transposon RND Efflux mutants.

	Antibiotic No Growth Zone (mm)	
***F. novicida***	**Avg**	**p-value**

wild-type	31.4 ± 1.0	

*ftlC*	28.0 ± 3.1	0.006

*tolC*	33.2 ± 1.4	0.007

*dsbB*	30.7 ± 1.2	0.162

*acrA*	23.5 ± 0.7	<0.001

*acrB*	25.2 ± 1.1	<0.001

***F. tularensis *Schu S4**	**Avg**	**p-value**

wild-type	25.5 ± 1.9	**--------**

Δ*acrA*	41.7 ± 2.7	0.0001

Δ*acrB*	35.7 ± 4.3	0.001

For *F. novicida *RND efflux mutants, 15 ug Az discs were from Remel, while for *F. tularensis *Schu S4, 15 ug Az discs were from Fluka. The zone of inhibition was measured in mm.

In the disc inhibition assay of the disulfide bond protein mutant *dsbB*, there was no significant difference compared to the wild-type (p-value = 0.162) (Table 6). Similarly, the MIC for *dsbB *was not significantly different than the wild-type value (p-value = 0.400) (Table 5). Thus, mutation of *dsbB *does not seem to have a significant impact on the ability of the organism to resist Az, whereas transposon insertion mutants in the *tolC, ftlC, acrA *and *acrB *components of the RND efflux system appear to decrease the sensitivity of *F. novicida *to Az. This result for *tolC *and *ftlC *may be in contrast to Gil et al. [12], who found that *F. tularensis *LVS deletion of *tolC *or *ftlC *did not alter the sensitivity to erythromycin (15 μg disc). The MIC of *F. tularensis *LVS is higher than can be achieved using a 15 μg disc, reported at >256 μg/ml erythromycin [28]. Therefore, any alteration in sensitivity due to *tolC *deletion would not be observed at this low concentration of antibiotic.

In contrast to the *F. novicida *results, the *F. tularensis *Schu S4 Δ*acrA *mutant and Δ*acrB *mutants had greater sensitivity to Az compared to the wild-type *F. tularensis *Schu S4 (p-value < 0.001) (Table 6). This is consistent with the findings of Qin *et al*. [16] who found an increased sensitivity of Δ*acrB *to 50 μg disc erythromycin. The MICs for Az against *F. tularensis *Schu S4 RND efflux mutants were also determined. The MICs for Δ*acrA *and Δ*acrB *(MIC > 1.5 μg/ml Az) are higher than the wild-type MIC of 0.78 μg/ml Az (p-value < 0.02) (Figure 4C, Table 5). However, the *F. tularensis *Schu S4 mutants for Δ*acrA *(EC_50 _of 0.085 μg/ml) and Δ*acrB *(EC_50 _0f 0.049 μg/ml) have EC_50_s less than the wild-type *F. tularensis *Schu S4 (EC_50 _of 0.145 μg/ml), reflecting the altered shape of the MIC curve and indicating increased sensitivity. Only Δ*acrB *was statistically significantly different for EC_50 _when compared to the wild-type *F. tularensis *Schu S4 (p-value < 0.05). Thus, *F. tularensis *Schu S4 Δ*acrA *and Δ*acrB *mutants had greater sensitivity to Az compared to *F. novicida *mutants, or the parental *F. tularensis *Schu S4 strain by disc inhibition assay and MIC.

### Az inhibition of intracellular *Francisella *mutant strains

**Figure 5:**
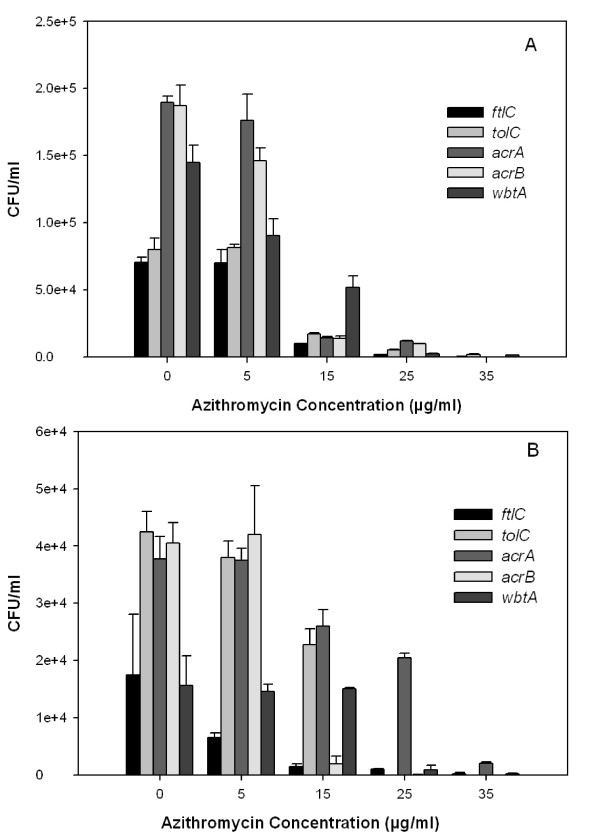
**Az inhibition of intracellular *F. novicida *mutants**. A) J774A.1 and B) A549 cells were infected with various mutants at an MOI 500. At 22 hours, the number of CFUs/ml recovered from *F. novicida *multidrug efflux mutants *ftlC, tolC, acrA*, and *acrB *and LPS O-antigen mutant *wbtA *decreased as Az concentrations increased and was near 0 CFU/ml at 35 μg/ml Az (p-value < 0.005 for all Az treatments compared to 0 μg/ml Az for each mutant). The recovery of mutant strains after Az treatments were significantly different from the wild-type *F. novicida *with a p-value < 0.05 (1.76 × 10^5 ^± 6.36 × 10^3 ^CFU/ml in J774A.1 at 0 μg/ml Az which decreased to 0 CFU/ml at 5 μg/ml Az and 1.80 × 10^5 ^± 1.41 × 10^4 ^CFU/ml in A549 cells at 0 μg/ml Az which decreased to 0 CFU/ml at 25 μg/ml Az). J774A.1 cells had higher bacterial counts than A549 cells.

J774A.1 and A549 cells infected with *F. novicida *transposon LPS mutant *wbtA *and multidrug efflux mutants *ftlC, tolC, acrA*, and *acrB *had more than 10^4 ^CFU/ml 22 hours post-infection (Figure 5). *ftlC *generally had lower CFU counts, whereas the *acrA *and *acrB *had higher CFU counts in both cell lines. The CFU of *F. novicida *transposon mutants decreased as the Az concentration increased for each cell line (p-value < 0.005 for each Az treatment compared to 0 μg/ml Az). At 35 μg/ml Az treatment, the bacterial CFU count was near 0 CFU/ml in J774A.1 and A549 cells (Figure 5). Thus, *wbtA *and the RND mutants are capable of replication within J774A.1 and A549 cells, although the overall number of bacteria per cell was lower than for the parental *F. novicida *infection (1.76 × 10^5 ^± 6.36 × 10^3 ^CFU/ml in J774A.1 and 1.80 × 10^5 ^± 1.41 × 10^4 ^CFU/ml in A549 cells at 0 μg/ml). Mutant trends after Az treatments were significantly different from the wild-type *F. novicida *with a p-value < 0.05 (wild-type decreased to 0 CFU/ml at 5 μg/ml Az in J774A.1 cells and decreased to 0 CFU/ml at 25 μg/ml Az in A549 cells). Corresponding to the higher MICs identified *in vitro*, LPS mutants require more Az to eliminate the bacteria from infected cells.

### G. mellonella infection by *Francisella *and antibiotic treatment

**Figure 6:**
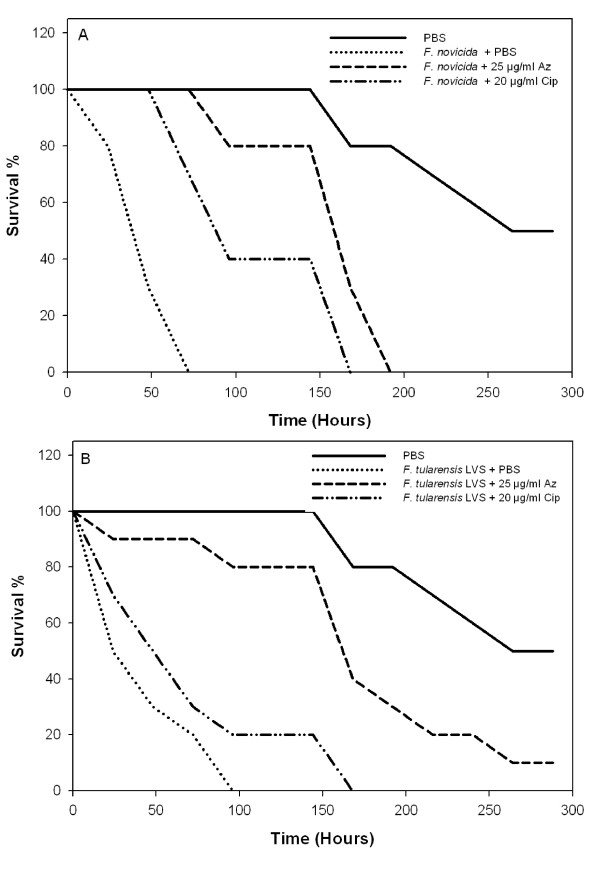
**Antibiotic treatment of *Francisella*-infected *G. mellonella***. High concentrations of antibiotics prolonged the survival of *G. mellonella *infected with 3 × 10^6 ^CFU *Francisella*. Non-infected control groups consisted of no injection, PBS injection, 25 μg/ml Az injection, or 20 μg/ml ciprofloxacin injection. All non-infected controls had similar high survival rates (data not shown for non-injected, 25 μg/ml Az injection, or 20 μg/ml ciprofloxacin injection). A) The infected control group received *F. novicida *injection, then PBS. A single dose of 25 μg/ml Az, given 2 hours after bacterial inoculation, was effective when compared to the infected control (p-value = 0.004). Treatment with 20 μg/ml ciprofloxacin prolonged the survival of the caterpillars compared to the control (p-value < 0.01). B) The infected control group received *F. tularensis *LVS injection, then PBS. A single dose of 25 μg/ml Az, given 2 hours after bacterial inoculation, was effective compared to the infected control (p-value < 0.001). Treatment with 20 μg/ml ciprofloxacin prolonged the survival of the caterpillars compared to control (p-value < 0.01). For *F. tularensis *LVS and *F. novicida *infections, survival time was longer in Az treated groups compared to ciprofloxacin treated groups (p-value < 0.02).

*Francisella-*infected *G. mellonella *was used as a model system [25] to study Az treatment. *G. mellonella *were infected with either 3 × 10^6 ^CFU bacteria/larva of *F. novicida *or *F. tularensis *LVS and then treated with a single dose of 10 μl injections PBS (no antibiotic), 20 μg/ml ciprofloxacin, or 25 μg/ml Az. Control groups (no infection) consisted of no injections, injections of either PBS (to measure trauma related to injections), 20 μg/ml ciprofloxacin, or 25 μg/ml Az (to assess antimicrobial agent effects on the host). All controls had similar survival rates (data not shown for antibiotic injection only controls). *Francisella*-infected *G. mellonella *did not survive past 100 hours post-infection. Control groups survived for more than 300 hours. Infected groups treated with a single dose 20 μg/ml ciprofloxacin (mean time to death > 74 hours) or 25 μg/ml Az (mean time to death > 160 hours) had a statistically significant prolonged survival times when compared to infected groups (p-value < 0.005) (Figure 6A &6B). These results are consistent with previously published results of *G. mellonella *infected with *F. tularensis *LVS and treated with 20 μg/ml ciprofloxacin [25]. Although we could not achieve complete recovery, *Francisella-*infected *G. mellonella *groups treated with Az had an increased mean survival time compared to ciprofloxacin-treated caterpillars (p-value < 0.02).

## Discussion

The macrolide erythromycin has limited efficacy against many gram-negative bacteria due to its hydrophobic nature and lack of permeability of the gram-negative outer membrane [31]. The sensitivity of erythromycin varies between *Francisella *strains. In the North American Type A *Francisella *strains, erythromycin MICs range from 0.5 to 4 μg/ml, while *F. tularensis *LVS has an MIC > 256 μg/ml [32]. The macrolide azithromycin is more effective against gram-negative bacteria than erythromycin [33]. Despite reports that European clinical strains of Type B *F. tularensis *are resistant to Az (MIC > 256 mg/L) [27], we observed that commonly used laboratory strains were sensitive to Az. In this study, we have demonstrated that the Type A *F. tularensis tularensis *strains are sensitive to Az *in vitro*. *F. philomiragia *and *F. novicida *are also sensitive with similar MICs. We determined that the MIC for *F. tularensis *LVS (NR-646) was 25 ug/ml Az, confirming the finding that LVS is relatively more resistant to Az than other *Francisella *strains.

Az is pumped out of gram-negative bacteria by several drug-efflux systems, including the RND efflux pumps. Az sensitivity differed between *F. novicida *and *F. tularensis *Schu S4 RND efflux mutants. Wild-type *F. tularensis *Schu S4 has similar sensitivity to Az as wild-type *F. novicida*, but the RND efflux mutants Δ*acrA *and Δ*acrB *in *F. tularensis *Schu S4 are more sensitive to Az, whereas the *F. novicida acrA *and a*crB *mutants are more resistant. These *F. tularensis *Schu S4 Δ*acrA *and Δ*acrB *mutants were also reported to be more sensitive to the related antibiotic erythromycin [16]. The difference between the *F. tularensis *Schu S4 and the *F. novicida *mutants might be due to the fact that *F. tularensis *Schu S4 has 254 pseudogenes; many of these genes are intact in *F. novicida *[34]. For example, in *F. tularensis *Schu S4, at least 14 genes of the MFS transporter superfamily contain stop codons or frameshifts [34,35] and are thus predicted to be non-functional. Additional types of transporter proteins, including a drug-resistance transporter (FTT1618), are also reported to be non-functional pseudogenes [34] in *F. tularensis *Schu S4. It could be that the remaining TolC-AcrAB pump is the major means by which *F. tularensis *Schu S4 pumps out Az. If this pump is compromised, the organism would be more susceptible to the antibiotic, because it may not have an operational alternative pump, such as the MFS or ABC transporters to pump out the drug. This is supported by the finding that Δ*acrA *and Δ*acrB *mutants in *F. tularensis *Schu S4 also displayed increased sensitivity to nalidixic acid (a substrate for the MFS transporter), as well as detergents, streptomycin, tetracycline, and other molecules [16]. In the case of *F. novicida*, there may be alternate systems that can pump out the drug in the absence of the RND system. Alternatively, the mutation in *acrA *or *acrB *may cause an up-regulation of expression of another drug-efflux pump, rendering the bacteria more resistant to the antibiotic [36,37]. Previous studies have shown that *dsbB *mutant in *F. tularensis *Schu S4 does not have any effect on antibiotic sensitivity (including the macrolide erythromycin) [16]. Consistent with the *F. tularensis *Schu S4 *dsbB *mutant, the *F. novicida dsbB *mutant showed no difference from the wild-type *F. novicida*.

Another common mechanism of resistance to macrolides is modification of the 23S rRNA. It has been reported that *F. tularensis *LVS has a point mutation in Domain V of the 23S rRNA, rendering it more resistant to erythromycin than *F. novicida *or *F. tularensis *Schu S4 [38]. This modification could also explain the increased resistance to Az in *F. tularensis *LVS. In addition, there are methylases that can confer increased resistance by targeted modification (methylation) of a specific adenine residue of the 23S rRNA. There are some methylases that have been identified as critical virulence factors for *Francisella *that might carry out this modification [39]. Some methylases that are present in the genome of *F. novicida *are either absent or are pseudogenes/nonfunctional genes (such as FTT0010, FTT0770, FTT1430, FTT1719, and FTT1735c) in *F. tularensis *Schu S4, potentially contributing to the different sensitivities to Az between the strains [34]. Any potential role of these molecules in Az sensitivity or resistance in *Francisella *has not yet been determined.

It has been suggested that Az attaches to the acidic LPS on the outer membrane of gram-negative bacteria, allowing the drug to penetrate through the outer membrane and enter the bacteria [40]. The *wbt *locus in *Francisella*, which is responsible for the production of LPS O-antigen, has been shown to be required for virulence [41]. In published reports, the *wbtA *mutant in *F. tularensis *LVS demonstrated a loss of the O-antigen and an inability to replicate in mouse macrophages. *F. novicida wbtA *mutants replicate normally and have only moderate sensitivity to serum [42,43]. We tested *F. novicida *transposon-insertion mutants *wbtN, wbtE, wbtQ *and *wbtA*, which are involved in the production of LPS, and found that these mutants were less susceptible to Az. Mutations of the LPS in the *F. novicida *transposon LPS O-antigen mutants may alter the LPS region presumed to bind to Az, resulting in a decreased amount of Az penetration and increased resistance to Az. Our results support the proposed role of LPS O-antigen in Az penetration into gram-negative bacteria such as *Francisella*.

Az is a weak base that can remain inside host cells for a longer time at a higher concentration than in the serum. This occurs because the basic amine groups of Az neutralize the lysosomal pH and prevent acidification of the lysosome. This process causes the drug to become trapped in the cell due to the positive charge. The drug is slowly released from polymorphonuclear neutrophils, allowing for a long half-life [8]. Az also concentrates in macrophages, which suggested to us that it might be useful as a potential treatment of intracellular pathogens such as *F. tularensis*. J774A.1 mouse macrophage were infected with *F. philomiragia, F. novicida*, and *F. tularensis *LVS and treated with Az. It was determined that 5 μg/ml Az was effective in eliminating intracellular *F. philomiragia, F. novicida*, and even *F. tularensis *LVS infections in J774A.1 cells. Although Type B strains are intrinsically more resistant to macrolides, *F. tularensis *LVS CFUs were eliminated below the Az MIC values for this strain. We suggest that J774A.1 cells can sufficiently concentrate Az so that the intramacrophage concentration of Az exceeds the MIC. Thus, it may be that Az is effective against LVS *in vivo *due to the concentration effect in macrophages. A concentration of 25 μg/ml Az was found to be effective against *Francisella *infections in A549 cells, suggesting that these non-phagocytic cells may be less able to concentrate the antibiotic intracellularly [22].

Az treatment has not been tested sufficiently in the clinic to know if it can be used to treat tularemia infection. In one reported case, the patient's illness was fatal after treatment by Az, trimethoprim-sulfamethoxazole, streptomycin, and ceftriaxone of *F. tularensis *[44], suggesting that the patient was extremely ill when treatment was initiated. In another case, the patient's symptoms decreased with a one day ceftriaxone treatment followed by a 5 day Az treatment, but symptoms recurred after the treatment was completed [45]. There have been several reports of successful treatment with erythromycin, giving credence to the sensitivity of Type A strains to the macrolide class of antibiotics [46,47]. To test the *in vivo *effectiveness of Az against *Francisella *infections, we employed the wax-moth caterpillar model [25]. The time-course of infection of the caterpillars closely matched the published report. We extended the published report by demonstrating that wax-moth caterpillars can also be infected by *F. novicida*. We demonstrated that a single injection of Az increased the mean survival time of *Francisella *infected *G. mellonella *and is more effective than a similar dose of ciprofloxacin. Within a host, macrolides, including Az, inhibit the production of cytokines that cause inflammation and prevent the accumulation of neutrophils, which suggests immunomodulatory effects separate from their antibacterial effects [48]. It has been shown that after *Francisella *infection in mice, there is a delayed response in the induction of host proinflammatory cytokines and recruitment of inflammatory cells to the site of infection, resulting in uncontrolled bacterial replication [49]. *G. mellonella*, however, does not have a similar immune response following *Francisella *infection. Since the therapeutic efficacy of Az cannot be observed in *G. mellonella*, future experiments will be conducted using a mouse model. Our results demonstrate efficacy of Az against multiple different *Francisella *strains and species. In future work, we will extend the Az studies to murine infections with the fully virulent strain, *F. tularensis *Schu S4.

## Conclusion

Az and other macrolide antibiotics may have a secondary benefit to patients with pneumonic tularemia infection since they also have immunomodulatory functions. Az has been used to treat non-infectious respiratory diseases such as diffuse panbronchiolitis (an inflammatory lung disease) and has been shown to reduce cytokine responses in the lungs thereby lessening the acute inflammatory response [48,50], even at sub-antimicrobial doses. Az is also used in the long-term management of lung transplant patients, including those with bronchiolitis obliterans syndrome, a disease occasionally resulting from the chronic immunological and inflammatory status in some post-transplant lungs [51]. Pulmonary tularemia often exhibits a robust pro-inflammatory response. If Az proves to be effective against *F. tularensis in vivo*, it may provide a dual therapeutic effect by also mitigating the pro-inflammatory response. Thus, there may be additional non-antimicrobial benefits to the lung as a result of using Az to treat pulmonary tularemia, which is often complicated by robust pro-inflammatory responses.

The current established treatment protocol for tularemia in children is ciprofloxacin [52]. However, ciprofloxacin has the potential for significant side effects, including liver toxicity, tendonitis and renal failure [40,53,54]. Az (trade name: Zithromax) is commonly prescribed to pediatric patients for ear infections and other common gram-negative infections, with very safe outcomes [55]. With the finding that Az concentrates in macrophages and is effective against *Francisella *species (including LVS)* in vitro *and in an *in vivo *infection model, we propose that further studies be done to establish the clinical utility of Az against tularemia, as an alternative treatment. In case of a deliberate tularemia infection of the population, such as in a biological weapons attack, there may be patients who can not tolerate the standard treatment. Az could be tested either as a stand-alone therapy or in combination with other chemotherapeutic agents. Developing an alternate effective therapy to treat tularemia in patients that do not tolerate ciprofloxacin well, such as pediatric and elderly patients, will lead to safer therapeutic options for physicians.

## Methods

### Antibiotics

The antibiotics investigated in this study were azithromycin (Az) (Biochemika), gentamicin (ATCC), and ciprofloxacin (Biochemika). Az was obtained as 15 μg discs (Fluka # 68601 or Remel # R33105), and dry powder (Fluka). Az was dissolved in distilled water and ciprofloxacin was dissolved in 0.5 M HCl to appropriate concentration. Gentamicin was obtained in solution at high concentration (50 mg/ml, ATCC) and diluted in distilled water.

### Bacterial strains

The following reagents were obtained through the NIH Biodefense and Emerging Infections Research Resources Repository, NIAID, NIH: *Francisella philomiragia *(ATCC #25015), *F. tularensis holarctica *Live Vaccine Strain (LVS) FSC155 (#NR-646), *F. novicida *(#NR-13), and *F. novicida *transposon insertion mutants (Table 7) [56]. Bacteria were grown in trypticase soy broth supplemented with cysteine (TSB-C) for 24 or 48 (for LVS, a slower growing organism) hours at 37°C in 5% CO_2 _to approximately 10^10 ^CFU/ml. *F. tularensis tularensis *strain NIH B38 (B38) (ATCC 6223; BEI Resources # NR50, deposited as the type strain for *F. tularensis tularensis*) was grown on Chocolate II Agar plates (BD Biosciences) at 37°C for 72 hours due to their extremely slow growth rate. LPS mutants in *wbtN, wbtE, wbtQ*, and *wbtA *loci were tested. RND efflux mutants in *dsbB, acrA, acrB, tolC*, and *ftlC *were also tested (Table 7). *F. tularensis *Schu S4 (CDC, Fort Collins, CO) and *F. tularensis *Schu S4 deletion mutants Δ*dsbB, *Δ*acrA*, and Δ*acrB *(21) were tested in an approved biosafety level 3 laboratory by trained personnel at the University of Virginia, Charlottesville, VA (Table 7).

**Table 7:** *F. novicida *and *F. tularensis *subsp. *tularensis *Schu S4 mutants used.

Mutant abbreviation	Mutant name	Gene
*wbtN*	tnfn1_pw060420p04q142	*wbtN *FTN_1422

*wbtE*	tnfn1_pw060328p03q164	*wbtE *FTN_1426

*wbtQ*	tnfn1_pw060419p04q158	*wbtQ *FTN_1430

*wbtA*	tnfn1_pw060419p03q166	*wbtA *FTN_1431

*tolC*	tnfn1_pw060419p03q111	*tolC *FTN_1703

tolC*	tnfn1_pw060328p03q137	*tolC *FTN_1703

*ftlC*	tnfn1_pw060418p04q166	Hypothetical protein FTN_0779

*dsbB*	tnfn1_pw060323p05q173	*dsbB *FTN_1608

*acrA*	tnfn1_pw060328p06q117	Membrane fusion protein FTN_1609

acrA*	tnfn1_pw060419p03q103	Membrane fusion protein FTN_1609

*acrB*	tnfn1_pw060323p02q131	RND efflux transporter, AcrB/AcrD/AcrF family FTN_1610

acrB*	tnfn1_pw060418p04q118	RND efflux transporter, AcrB/AcrD/AcrF family FTN_1610

Δ*acrB*	BJM1032	Schu S4 Δ*acrB *[16] (FTT0105c)

Δ*acrA*	BJM1040	Schu S4 Δ*acrA *[16] (FTT0106c)

(*= these mutants were tested, but data is not shown as it was the same as the first mutant).

### Cell culture

Mouse macrophage cells J774A.1 (ATCC #TIB-67) and human lung epithelial cells A549 (ATCC #CCL-185) were obtained from ATCC, Manassas, VA. J774A.1 cells were grown in Dulbecco's Modified Eagle Medium (DMEM) with 10% fetal bovine serum and passed every 3 days in a 1:3 dilution following manufacturers' instructions. A549 cells were grown in Ham's F-12 with 10% fetal bovine serum and passed every 3 days in a 1:3 dilution.

### Disc inhibition assay

Kirby-Bauer disc inhibition assay protocol was followed [57]. 100 μl of overnight bacterial cultures were spread on Chocolate II agar and Schu S4 strains were spread on Mueller-Hinton agar plate with three discs each containing 15 μg Az placed in a triangle and incubated based on length of time for bacterial growth to be seen on the plate: 24 (for *F. novicida, F. philomiragia*, and *F. tularensis *Schu S4), 48 (for *F. tularensis *LVS), and 72 hours (for *F. tularensis *NIH B38) at 37°C in 5% CO_2_. The diameter of the zone of inhibition including the 6 mm disc was measured (in mm) with three independent measurements for each zone (n = 9). Inhibition was defined as the area of no bacterial growth around the discs. A reading of 6 mm indicates no inhibition [57].

### Minimal inhibitory concentration (MIC)

Assays were performed with small modification following published protocols [58]. The MIC for *F. novicida, F. philomiragia, F. tularensis *LVS, related *F. novicida *mutants, *F. tularensis *Schu S4, and related *F. tularensis *Schu S4 mutants were determined in TSB-C media by antibiotic dilution in triplicates. The broth was then inoculated with 10^5 ^CFU/ml per strain. Concentration of the antibiotics ranged from 1 mg/ml to 0.0001 μg/ml. The MIC was read at optical density 600 nm after 24 hours (for *F. philomiragia, F. novicida*, and *F. tularensis *Schu S4) and after 48 hours (for *F. tularensis *LVS) and was defined as the lowest concentration of antibiotic with no visible growth.

### Data analysis and statistics

Data were analyzed using the following equation and GraphPad Prism 4 (GraphPad Software Inc., San Diego, CA) [23].(1)

Y corresponds to bacterial mortality (% OD, where zero drug = 100%) at a given antibiotic concentration (μg/ml), with X being the logarithm of that concentration (log μg/ml). In the equation, "Top" and "Bottom" refer to the upper and lower boundaries, and were constrained to values <100% and >0%, respectively. EC_50 _values were determined by fitting the data from the antimicrobial assays to a standard sigmoidal dose-response curve (Equation 1) with a Hill slope of 1. Control samples with no antibiotic are plotted as 10^-4 μg/ml for graphing purposes. Errors were reported based on the standard deviation from the mean of the Log EC_50 _values. Student's T-test was used to determine whether points were statistically different, using a two tailed test assuming normal distribution.

### Cell infection with *Francisella *strains

J774A.1 cells and A549 cells were plated (10^5^/well) in a 96-well plate and infected with either *F. novicida, F. philomiragia, F. tularensis *LVS, or *F. novicida *transposon mutants at MOI 500 for 2 hour incubation. Extracellular bacteria were removed by washing cell wells twice with DMEM for J774A.1 cells or Ham's F-12 for A549 cells. After *Francisella *infection and removal of extracellular bacterium, cells were incubated with 50 μg/ml gentamicin for 1 hour to eliminate extracellular bacterium but which does not affect intracellular bacteria. Cells were washed with media twice and incubated with Az in the media at final concentrations of 0, 0.1, 5, 15, 25, and 35 μg/ml for 0 or 22 hours at 37°C.

### Quantification of intracellular *Francisella *bacteria

After exposure of cells to *Francisella *and antibiotics, the numbers of intracellular bacteria were determined. At 0 and 22 hours, the samples were washed twice with PBS. Sterile deionized water was used to lyse cells. Aliquots of cells and cell-associated bacteria were serially diluted onto chocolate agar plates, incubated at 37°C and 5% CO_2 _for 1 or 2 days and the CFU were counted.

### Quantification of cellular apoptosis

After exposure of cells to *Francisella *and antibiotics, the numbers of cell-associated bacteria were determined, the CytoTox-96^® ^Non-radioactive Cytotoxicity Assay (Promega) was used to quantitatively measure lactate dehydrogenase (LDH) release at 22 hours, following manufacturers' instructions. Absorbance values were recorded at OD 490 nm by spectrophotometer (μQuant, BioTek). Background noise values were subtracted from sample readings. Determine % cell death using formula:(2)

### Galleria mellonella exposure to *Francisella *strains and treatment with antibiotics

*Galleria mellonella *was obtained at the larval stages from Vanderhorst Wholesale (Saint Marys, OH). 10 caterpillars with a weight of 0.30-0.35 g were used for each group. Injection area was cleaned with water and a 10 μl Hamilton syringe was used to inject 10 μl of 3 × 10^6 ^CFU/ml of either *F. novicida *or *F. tularensis *LVS into the hemocoel of each caterpillar via the last left proleg and incubated at 37°C for 2 hours [25]. Caterpillars were then injected with 10 μl of either PBS, 25 μg/ml Az, or 20 μg/ml ciprofloxacin in the last right proleg. Control caterpillars were either not injected or injected with only PBS, azithromycin, or ciprofloxacin. Caterpillar groups were incubated at 37°C and scored daily for color change or death.

## Authors' contributions

SA carried out the cell-based assays, the *in vitro *studies with the mutants and the caterpillar experiments, analyzed the data and contributed to writing the manuscript. LH conceived the original use of Az against intracellular *Francisella *and performed the first *in vitro *studies of Az's effectiveness, AQ performed the Schu S4 testing, BM designed and coordinated the Schu S4 testing and contributed to the interpretation and conclusions drawn from these studies, MVH conceived of the overall study, designed and coordinated the experiments, and wrote the manuscript. All authors read and approved the final manuscript.
